# Management of acute fever in children: Consensus recommendations for community and primary healthcare providers in sub-Saharan Africa

**DOI:** 10.1016/j.afjem.2020.11.004

**Published:** 2021-04-10

**Authors:** Robin Green, David Webb, Prakash Mohan Jeena, Mike Wells, Nadia Butt, Jimmy Mapenzi Hangoma, Rajatheran (Sham) Moodley, Jackie Maimin, Margreet Wibbelink, Fatima Mustafa

**Affiliations:** a Department Paediatrics and Child Health, University of Pretoria, South Africa; b Houghton House Group, Johannesburg, South Africa; c Department of Paediatrics & Child Health, University of KwaZulu Natal, Durban, South Africa; d Division of Emergency Medicine, University of the Witwatersrand, Johannesburg, South Africa; e Health Aid Chemist, Nairobi, Kenya; f Levy Mwanawasa Medical University, School of Health Sciences, Lusaka, Zambia; g Care Group of Pharmacies, South Africa; h South African Pharmacy Council, Johannesburg, South Africa; i Sister Lilian Centre (Pty), Pretoria, South Africa; j Steve Biko Academic Hospital, Department of Paediatrics and Child Health, University of Pretoria, South Africa

**Keywords:** Childhood, Fever, Febrile, Primary healthcare, Sub-Saharan Africa

## Abstract

Fever is one of the most common reasons for unwell children presenting to pharmacists and primary healthcare practitioners. Currently there are no guidelines for assessment and management of fever specifically for community and primary healthcare workers in the sub-Saharan Africa region. This multidisciplinary consensus guide was developed to assist pharmacists and primary healthcare workers in sub-Saharan Africa to risk stratify and manage children who present with fever, decide when to refer, and how to advise parents and caregivers.

Fever is defined as body temperature ≥ 37.5 °C and is a normal physiological response to illness that facilitates and accelerates recovery. Although it is often associated with self-limiting illness, it causes significant concern to both parents and attending healthcare workers. Clinical signs may be used by pharmacy staff and primary healthcare workers to determine level of distress and to distinguish between a child with fever who is at high risk of serious illness and who requires specific treatment, hospitalisation or specialist care, and those at low risk who could be managed conservatively at home. In children with warning signs, serious causes of fever that may need to be excluded include infections (including malaria), non-infective inflammatory conditions and malignancy. Simple febrile convulsions are not in themselves harmful, and are not necessarily indicative of serious infection. In the absence of illness requiring specific treatment, relief from distress is the primary indication for prescribing pharmacotherapy, and antipyretics should not be administered with the sole intention of reducing body temperature. Care must be taken not to overdose medications and clear instructions should be given to parents/caregivers on managing the child at home and when to seek further medical care.

## African relevance

• Countries in sub-Saharan Africa face healthcare challenges unlike those in Western countries.
• Currently there are no guidelines for assessment and management of fever specifically for community and primary healthcare workers in the sub-Saharan Africa region.
• Available published fever guidelines have been written for middle-upper income and/or Western countries.
• This multidisciplinary consensus guide was developed to assist pharmacists and primary healthcare workers in sub-Saharan Africa to risk stratify and manage children who present with fever, decide when to refer, and how to advise parents and caregivers.

This consensus document is divided into two sections. Section A is a quick reference guide containing the main tables and recommendations relevant to the primary healthcare practitioner nurse and pharmacy staff. Section B contains the complete document.

## Section A. Quick reference guide for primary healthcare practitioner, nurse and pharmacy staff

• Fever is defined as axillary temperature ≥ 37.5 °C.
• Fever in itself is not life-threatening, but rather a normal physiological response to infection or illness.
• Most fevers are due to a self-limiting viral infection, but bacterial infections, malaria and other causes must be ruled out.
• Body temperature should be measured in the axilla, or in the ear using an infrared tympanic thermometer (after 4 weeks of age).

Refer the child to a doctor if:

1. Child is younger than 3 months of age.
2. Child is not eating or drinking normally.
3. Child is not behaving normally; e.g.,
  • Nonresponsive
  • Lethargic
  • Persistent, inconsolable crying, or cries when touched.
4. Fever has been present for longer than 2 days (48 hours).
5. Temperature higher than 40 °C.
6. Persistent vomiting.
7. Abnormal breathing.
8. Convulsions.
9. Non-blanching rash.
10. Urine is very dark in colour, and/or the child cries when passing urine.
11. Swelling of a limb or joint.
12. Child looks very ill or has signs of dehydration or shock:
  • Sunken fontanelle
  • Dry mouth
  • Absence of tears
  • Sunken eyes
  • Poor overall appearance.
13. Danger signs associated with severe malaria:
  • Impaired consciousness
  • Generalised weakness
  • Multiple convulsions
  • Acidosis
  • Hypoglycaemia
  • Severe malarial anaemia (Hb ≤5 g/dL or haematocrit ≤15% in children age <12 years with a parasite count of >10 000/uL)
  • Renal impairment
  • Jaundice
  • Pulmonary oedema
  • Significant bleeding
  • Shock
  • Hyperparasitaemia (*P. falciparum* parasitaemia >10%; *P. knowlesi* >100 000/uL or jaundice and >20 000/uL; *P. vivax* has no density threshold)

**For children with less severe illness:**

**Advise parents/caregivers about when to seek medical care for their child**

• Any of the danger signs listed above.
• If the parent is worried for any other reason.

**Antipyretic medication**

• Either paracetamol or ibuprofen (not both) can be used to make the child more comfortable.
• Dose of antipyretic medication for infants and children older than 3 months of age:
  ○ Paracetamol: 15 mg/kg body weight (up to 1 g) every 6 h as necessary (maximum daily dose is 90 mg/kg or 4 g in total)
  ○ Ibuprofen: 10 mg/kg body weight every 6 h as necessary (maximum daily dose is 40 mg/kg)

**Table:** 

Dosing by body weight for oral liquid: Ibuprofen 100 mg/5 mL every 6 h	
Body weight (kg)	Using a syringe (mL)
6	3.0
9	4.5
12	6.0
15	7.5
18	9.0
21	10.5
24	12.0
27	13.5
≥30	15.0
Do not exceed 40 mg/kg per day	

Dosing by body weight for oral liquid: Paracetamol 120 mg/5 mL every 6 h	
Body weight (kg)	Using a syringe (mL)
6	3.5
9	5.5
12	7.5
15	9.5
18	11.0
21	13.0
≥24	15.0
Do not exceed 90 mg/kg or 4 g in total per day	

**General advice for parents and caregivers**

1. Reassure parents with anxiety about the child's fever.
2. Advise the parent on management of fever at home (see below).
3. Give clear instructions on how to administer medication.
4. Advise parents on the correct use of paracetamol or ibuprofen.
5. Provide written instructions about when to come back if the child's condition gets worse.

**Table:** 

Advice for parents (and caregivers) about management of fever at home	
Do	Don't
**Do** encourage the child to drink fluids regularly (breast milk is best for breast feeding children). **Do** make sure medication is given in the right dose at the right time. **Do** seek further medical advice if the fever does not get better within 48 h, or if the child's condition gets worse.	**Don't** over-dress or under-dress the child, or wrap the child in heavy blankets. **Don't** allow children to drink medicines straight from the bottle. **Don't** administer another dose of antipyretic medication immediately if the temperature does not come down after one dose. Wait for the appropriate dosing interval to pass and only give another dose at the correct time. **Don't** wake a sleeping child just to administer antipyretic medication. **Don't** give antipyretic medicine to the child for longer than 2 days without consulting a doctor.

**Malaria**

• Malaria must be considered in all children with fever who live in or who have recently travelled to an endemic area.
• Local guidelines should be consulted to guide management and prescription of appropriate antimalarial medication.
• Severe malaria is a medical emergency. Children with severe febrile disease should be given a first dose of an antimalarial drug and an antibiotic, and referred immediately to hospital.
• Children without severe illness and who test positive for malaria can be treated at the clinic with follow-up as necessary.

## Section B. Full guideline

### Introduction

Fever is a normal physiological response to illness that facilitates and accelerates recovery [1]. There is no evidence that children with fever are at increased risk for adverse outcomes, though it is frequently a cause for concern among both parents and healthcare providers who fear it may be associated with increased morbidity, such as seizures, brain damage or death [2,3]. There is confusion about how and whether to manage fever, and antipyretics are frequently prescribed or purchased over-the-counter (OTC) specifically to bring down body temperature in an ill child [[3], [4], [5], [6]]. In many parts of the world, including Africa, paracetamol and nonsteroidal anti-inflammatories (NSAIDs; e.g., ibuprofen) are the most frequently purchased OTC or prescribed medicines for children, but inappropriate and incorrect use (wrong dose and/or time interval of administration) is common [4,[7], [8], [9], [10], [11], [12]].

Fever in children is one of the most common reasons for parents or caregivers to seek medical attention for their child [13,14]. Although fever is often a presenting symptom of a self-limiting viral infection, it is also associated with serious viral, bacterial and parasitic infections. It also occurs with non-infective inflammatory conditions (e.g., juvenile chronic arthritis) and cancer (e.g., acute leukaemia). Antibiotics are often inappropriately prescribed, exposing the child to unnecessary treatment-related adverse effects and increased risk of antibiotic resistance [[15], [16], [17]]. Misdiagnosis leads to inappropriate prescribing. Therefore, the underlying illness causing the fever needs to be determined and it is essential to distinguish between a child with fever who is at high risk of serious illness and who requires specific treatment, hospitalisation or specialist care, and one at low risk who can be managed conservatively at home. This is not only true for clinicians, but also for pharmacists and pharmacy support personnel, who are often the first to see the ill child or caregiver.

In 2013, the South African Fever group published a guideline for assessment and management of childhood fever in primary care [18]. Since then new studies, international guidelines [[19], [20], [21], [22], [23], [24], [25], [26]] and commentaries [27] on those have been published, warranting a revision of the 2013 guideline. Furthermore, we recognised that we share a number of challenges with our northern colleagues and to date, as far as we are aware, there has been no specific guideline for the sub-Saharan Africa region in general.

This multidisciplinary consensus guide to assessment and management of fever in children has been developed to assist pharmacists and primary healthcare workers in sub-Saharan Africa to risk stratify and manage children who present with fever, decide when to refer, and how to advise parents and caregivers.

### Methods

PubMed and Google searches were performed to identify published international guidelines, studies and reviews related to management of childhood fever to compile an updated guidance document. The primary keywords used for the search included ‘fever’, ‘children’, ‘paediatric’, ‘pediatric’, ‘guideline’, ‘antipyretic’, ‘ibuprofen’, ‘paracetamol’, ‘acetaminophen’, ‘mefenamic acid’, and ‘malaria’. We paid particular attention to publications dated 2013 and later. Thereafter, the previous guideline was updated and expanded by two of the authors (DW and RG). That document and successive drafts were reviewed and revised by a multidisciplinary group of pharmacists and clinicians from South Africa, Kenya and Zambia based on available evidence and agreement from their own clinical experience in their country. Standard levels of evidence were assigned to support the recommendations as follows: A, at least two randomised trials; B, single randomised clinical trial or large nonrandomised studies; C, consensus opinion of the experts based on observational studies and clinical experience. Therefore, the guidance here is based on consensus best practice across different disciplines and does not constitute inflexible treatment recommendations.

Because the guidance is specifically for healthcare providers, patient and community representatives were not directly involved, although community pharmacists and a primary care nursing professional with experience in clinics for mothers and young children were represented among the authors.

#### What is fever? (Evidence A)

• The World Health Organisation (WHO) defines fever as an axillary temperature ≥ 37.5 °C [20].
• Fever in itself is not detrimental, but rather a normal physiological response to infection or illness. In the absence of a diagnosis, treatment with the sole aim of reducing temperature is inappropriate and some febrile children will recover more quickly if the fever is not treated [2,3].
• Most fevers are due to a self-limiting viral infection, but bacterial infections, malaria and other causes must be excluded [21].
• Serious bacterial infection is more common in infants <1 year of age.
• Any fever in an infant aged <3 months is significant and should be thoroughly investigated and referred to specialist care or hospital if the source of the fever cannot be found.

#### Measurement of body temperature (Evidence A)

• Body temperature should be measured in the axilla, or in the ear using an infrared tympanic thermometer (only after 4 weeks of age).
• Oral and rectal routes should not be used to measure body temperature in a child.
• Re-usable thermometers should be appropriately cleaned between uses.
• The temperature reading may be inaccurate if the child is wearing a lot of clothing or is wrapped in a blanket. Under these circumstances they should be allowed to cool down before taking the temperature.

##### Axillary measurement

• Do not measure temperature directly after bathing.
• Ensure that the child's axilla is dry.
• If using a glass thermometer, before placing it in the axilla, shake the thermometer until the liquid is at or below the 36 °C line.
• Place the tip of the thermometer in the armpit and lightly press the child's elbow against the chest to close the tip of the thermometer in the armpit.
• Read the temperature:
  ○ If using a glass thermometer: after 4 min.
  ○ If using a digital thermometer: when the indicator sound (‘beep’) is heard.

##### Infra-red tympanic (ear) measurement

• Attach a new lens filter to the tip of the thermometer each time it is used.
• Turn the thermometer on.
• Gently pull backwards (posteriorly) on the ear lobe to open the ear canal.
• Insert the probe of the thermometer into the ear canal and press the activation button until the beep is heard.
• Remove the thermometer from the ear and read the temperature on the LCD screen.

##### Non-contact infrared thermometers

• The use of noncontact thermometers in children with fever is not recommended.
• Noncontact thermometers that measure temperature when held a few centimetres from the skin (e.g., forehead) can have a good negative predictive value and may be helpful in screening to exclude fever in a nonfebrile child.
• In children with fever, these devices are often unreliable and do not necessarily provide an accurate reading of body temperature when compared with axillary or tympanic measurements [[28], [29], [30], [31], [32], [33], [34]]. The device must be properly calibrated, and readings may vary depending on the child's level of distress, the distance between the handheld device and the child's head, and the number of readings taken.

#### Assessment by the primary healthcare practitioner (HCP) nurse and pharmacy staff

The majority of children with fever in Africa are seen primarily by pharmacists, pharmacists' assistants or nurses. Although in most cases fever is caused by a self-limiting illness, it can be a symptom of severe illness and care must be taken to identify children who require referral to a medical practitioner. Clinical features that indicate need for immediate referral are listed in Box 1.

Box 1 **Danger signs in a child with fever mandating immediate referral to a medical practitioner**

1. Child is younger than 3 months of age.
2. Child is not eating or drinking normally.
3. Child is not behaving normally; e.g.,
  • Nonresponsive
  • Lethargic
  • Persistent, inconsolable crying, or cries when touched.
4. Fever has been present for longer than 2 days (48 hours).
5. Temperature higher than 40 °C.
6. Persistent vomiting.
7. Abnormal breathing.
8. Convulsions.
9. Non-blanching rash.
10. Urine is very dark in colour, and/or the child cries when passing urine.
11. Swelling of a limb or joint.
12. Child looks very ill or has signs of dehydration or shock:
  • Sunken fontanelle
  • Dry mouth
  • Absence of tears
  • Sunken eyes
  • Poor overall appearance.

Alt-text: Box 1

##### Advise parents/caregivers about when to seek medical care for their child

A child who does not appear to be ill, who is staying awake and alert, has a strong cry or is not crying, or is smiling, is unlikely to have a serious cause of fever [35]. However, parents should be advised to seek immediate medical care if the child's condition worsens, if the child develops any of the danger signs listed in Box 1, or if the parent is worried for any other reason. If possible, parents should be given written information to take home with them.

#### Medical history and examination by a medical practitioner

Fever is not a diagnosis, but a symptom of illness. A diagnosis of the underlying illness is essential to institute appropriate treatment.

##### History

Ask about:

• Fever: onset, duration, continuous or intermittent, response to general measures or medication.
• Associated signs and symptoms.
• Recent use of antibiotics.
• Recent vaccinations (within 48 h) and vaccination history.
• Recent travel (especially to an area where malaria is endemic).
• Health of other family members, exposure to sick individuals, crèche/school.
• Previous illnesses (including recurring symptoms, immunodeficiency and chronic illnesses).
• Activity level, including joint or body pains and bruises.

##### Examination

A complete examination is mandatory in all children presenting with fever, with identification of symptoms and signs that predict risk of serious illness (Table 1). Depending on geographical region, attention should be paid to possible sources of infection (Table 2). In particular, malaria and measles, two important causes of childhood mortality, must be excluded [20].

**Table 1:** Symptoms and signs indicating low, intermediate and high risk for serious illness [21] (Evidence A).

	Low risk	Intermediate risk	High risk
Age		• Age 3–6 months with temperature ≥ 39 °C	• Age < 3 months with temperature ≥ 38 °C
Colour	• Normal colour of skin, lips and tongue	• Pallor of skin, lips or tongue reported by parent or carer	• Pale/mottled/ashen/blue skin, lips or tongue
Activity	• Responds normally to social cues • Content/smiles • Stays awake or awakens quickly • Strong normal cry or not crying	• Not responding normally to social cues • No smile • Wakes only with prolonged stimulation • Decreased activity • Poor feeding in infants	• No response to social cues • Appearing ill to a healthcare professional • Does not wake or if roused does not stay awake • Weak, high-pitched or continuous cry
Respiratory		• Nasal flaring • Respiratory rate >50 breaths/min (age 1–12 months) or >40 breaths/min (age > 12 months) • O_2_ saturation ≤ 95% in air • Crackles in chest	• Grunting • Respiratory rate >60 breaths per minute • Moderate or severe chest indrawing
Circulation and hydration	• Normal skin and eyes • Moist mucous membranes • Passing urine adequately	• Heart rate >160 beats/min (age < 12 months) or >150 beats/min (age 12–24 months) or >140 beats/min (age 2–5 years) • Capillary refill time ≥ 3 s • Dry mucous membranes • Reduced urine output	• Reduced skin turgor
Other	• No intermediate or high risk factors present	• Fever >5 days • Rigors • Swelling of a joint or limb • Non-weight bearing limb or not using an extremity	• Non-blanching rash • Bulging fontanelle • Neck stiffness • Status epilepticus • Focal neurological signs • Focal seizures

Adapted with permission from ©NICE (2019). NG143 Fever in under 5 s: assessment and initial management. Available from http://www.nice.org.uk/guidance/ng143. All rights reserved. Subject to Notice of rights. NICE guidance is prepared for the National Health Service in England. All NICE guidance is subject to regular review and may be updated or withdrawn. NICE accepts no responsibility for the use of its content in this publication.

**Table 2:** Important infectious causes of fever that must be excluded [2,20,21,41].

• Malaria • Bone and joint infections • Dengue haemorrhagic fever • Dysentery, enteritis • Herpes simplex encephalitis • HIV • Kawasaki disease • Measles • Chicken pox (varicella zoster virus) • Paramyxovirus	• Meningitis • Otitis media • Pneumonia • Septicaemia/bacteraemia • Sinusitis • Skin & soft tissue infections (e.g., impetigo, cellulitis) • Streptococcal pharyngitis • Tuberculosis • Typhoid • Urinary tract infection

##### COVID-19

Fever is a common presenting symptom in children with coronavirus (SARS-CoV-2) disease 2019 (COVID-19). According to information available at the time of writing, the infection rate in children is unknown. However, in comparison with adults, COVID-19 has been uncommon in children (approximately 1–5% of all cases), with a milder disease course and better prognosis [36,37]. Comorbidities appear to be an important factor in the rare cases who do develop severe disease [38,39]. In addition, a paediatric multisystem inflammatory syndrome (PMIS) with some features similar to Kawasaki disease (vasculitis) and toxic shock syndrome, and which may lead to multiorgan failure and shock, has recently been described in children and adolescents with COVID-19 requiring admission to intensive care units [37]. Where SARS-CoV-2 infection is a possibility, relevant guidelines should be consulted for detailed guidance on assessment and management.

##### Malignancy

Malignancy is a rare cause of fever and unnecessary investigations should be avoided. Nonspecific signs and symptoms that could be associated with an underlying malignancy (especially if recurring or persistent) are listed in Table 3 and need to be interpreted in combination with other findings on history and examination [40].

**Table 3:** Red flags associated with childhood malignancies [40].

• Pallor, fatigue, malaise • Fever • Anorexia and weight loss • Lymphadenopathy • Vomiting • Headaches • Recurrent or treatment-resistant infections • Bone pain, joint pain, refusal to walk • Back pain	• Urine retention, enuresis • Palpable abdominal mass • Hepatosplenomegaly • Scrotal swelling or mass • Gingival swelling/bleeding • Masses or lumps on extremities, head, neck, trunk • Bleeding • Skin conditions not responding to conventional treatment

#### General principles of management *(Evidence A)*

##### General principles

• Infants age < 3 months with temperature ≥ 38 °C are considered high risk, and infants age 3–6 months with temperature ≥ 39 °C are considered intermediate risk for serious illness [21].
• However, in general, the level of fever (height of body temperature) is not an accurate independent measure of severity of illness in febrile children and cannot be used to distinguish between bacterial and viral infection [[42], [43], [44]].
• In areas where malaria is endemic, children presenting with fever should be tested for malaria (see *Malaria*).
• Infants 2 months of age or younger who have a recent history of fever, but who are afebrile at presentation are still at increased risk of serious bacterial infection and require further investigation [45,46].
• Duration of fever should not be used to predict likelihood of serious illness [13,21,47].
• Urine analysis (dipstick test strip positive for nitrite and/or leucocyte esterase) is a simple and inexpensive test that increases detection of urinary tract infections in febrile young children and should be considered to complement clinical assessment described in Table 1 [48].
• Do not administer antibiotics unless there is clear evidence of bacteraemia [2].
• Provide general advice (Box 1, Box 4, [5]) for all parents, especially those who are unlikely to return for follow-up (because of, e.g., lack of transport, parental perception that the child is not that ill, no telephone).

##### Children classified as high risk

• High risk children with immediate life-threatening illness and neonates younger than 1 month of age should be referred to hospital or specialist care.
• Before transfer to hospital or specialist care ensure respiratory and haemodynamic stability and treat hypoglycaemia.
• If time from referral to hospital is >1 h, and it is indicated, give first dose of an appropriate intramuscular antibiotic.
• Children who are expected to be in the hospital emergency department for >1 h should have their first IV dose of antibiotic as soon as it is decided that it is needed; i.e., before going to the ward or high acuity unit.
• In areas where malaria is endemic, perform a rapid test to confirm presence of malaria. Where malaria is suspected and immediate diagnostic testing is not possible, or if the test is positive, administer the first dose of antimalarial medication. Where necessary, refer urgently to hospital (see *Malaria*).

##### Children classified as low risk

• Reassure the parents/caregiver and advise them on home management.
• Advise follow-up if symptoms do not resolve within 24–48 h.
• Provide general advice on when to seek further medical help (Box 1).
• Written instructions are preferable.

##### Antibiotics *(Evidence A)*

Inappropriate prescribing of broad spectrum antibiotics is associated with alterations in the microbiome (dysbiosis), selection for drug-resistant bacteria and rapid development of bacterial resistance in both the individual patient and in the community [[49], [50], [51]]. Disruption of the microbiota allows for overgrowth of pathogenic microorganisms with the attendant risks of antibiotic-associated diarrhoea, pseudomembranous colitis and *Candida* infection [[50], [51], [52], [53], [54]]. In some epidemiological studies, dysbiosis in early childhood has been positively associated with increased risk of disease in later life including atopy (increased tendency to asthma, allergic rhinitis and atopic eczema), obesity, diabetes and inflammatory diseases [50,55].

• Fever in itself is not an indication for antimicrobial therapy and antibiotics should only be prescribed if there is a clear indication that they are warranted.
• The majority of upper respiratory tract infections (URTI) are of viral origin, and bacterial URTIs are frequently self-limiting and resolve spontaneously. Most children with simple otitis media or tonsillitis do not require an antibiotic [56].
• Therefore, especially in patients without severe disease and where adequate follow-up is available, consider deferring antibiotic therapy for 48 h while symptomatic therapy is administered [56,57].
• Where necessary and feasible, microbial samples should be taken to inform prescribing should it be decided that antibiotics are justified [57].
• Empiric antibiotics for possible occult bacteraemia in infants >3 months of age do not confer any significant advantage [58,59]. However, young children at risk should be closely monitored.
• If the child has signs of severe sepsis or septic shock, parenteral antibiotics should be administered within the first hour of healthcare contact.
• Local guidelines and laboratories for microbial antibiotic sensitivities should be consulted to inform antibiotic prescription.
• Cases where antibiotics should be considered are listed in Table 4.

**Table 4:** Children with upper respiratory tract infection who require consideration for antibiotics [56].

• Complicated and/or severe initial presentation • Prevention of rheumatic heart disease (group A β-haemolytic streptococcal pharyngotonsillitis) • Acute otitis media • Immunocompromised patients • Neonates (child younger than 28 days) • Structural ENT or immunological abnormalities • Patients with limited access to healthcare • Acute bacterial sinusitis • Clinical deterioration on supportive therapy

##### Malaria

Although malaria is not widespread in South Africa, it is endemic in many other regions of sub-Saharan Africa. Therefore, it is an important differential diagnosis that must be considered in all children with fever who live in or who have recently travelled to these areas. Local guidelines should be consulted to guide management and prescription of appropriate antimalarial medication.

**Fig. 1:**
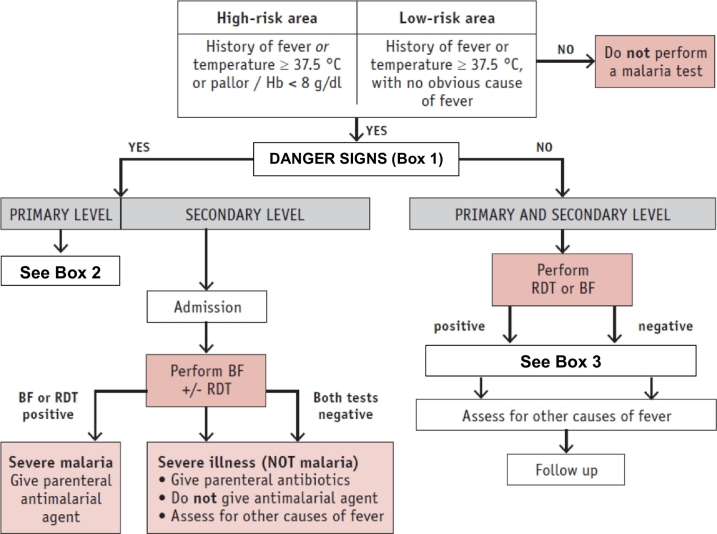
Approach to management of a child with fever who is at risk of malaria. Hb: haemoglobin; BF: blood film; RDT: rapid diagnostic test. Adapted with permission from World Health Organisation (WHO). WHO Informal Consultation On Fever Management In Peripheral Health Care Settings. A Global Review Of Evidence And Practice. Geneva, WHO Press; Copyright (2013). p16. https://www.who.int/malaria/publications/atoz/9789241506489/en/.

Signs and symptoms of malaria are nonspecific and similar to those of other febrile illnesses, so where there is suspicion of malaria, a blood film or other diagnostic test must be performed to confirm or exclude infection. Severe malaria is a medical emergency. Children with severe febrile disease (Box 1 and Table 5) should be given a first dose of an antimalarial drug (preferably artesunate) and an antibiotic, and referred immediately to hospital. If artesunate is unavailable or contraindicated, artemether or quinine are alternative antimalarial options. Children without severe illness and who test positive for malaria can be treated at the clinic with follow-up as necessary (Fig. 1).

**Table 5:** Danger signs associated with severe malaria [61].

• Impaired consciousness • Generalised weakness • Multiple convulsions • Acidosis • Hypoglycaemia • Severe malarial anaemia (Hb ≤5 g/dL or haematocrit ≤15% in children age < 12 years with a parasite count of >10,000/uL) • Renal impairment • Jaundice • Pulmonary oedema • Significant bleeding • Shock • Hyperparasitaemia (*P. falciparum* parasitaemia >10%; *P. knowlesi* > 100,000/uL or jaundice and > 20,000/uL; *P. vivax* has no density threshold)

Box 2 **Management of child in primary care at risk of malaria and with very severe febrile disease [20,61] (Evidence A).**

**Table:** 

• If available, perform a rapid test to confirm presence of malaria. • Give first dose of artesunate for severe malaria.^a^ • Give first dose of an appropriate antibiotic. • Treat the child to prevent low blood sugar. • Give one dose of paracetamol or ibuprofen in clinic for high fever (≥38.5 °C). • Refer urgently to hospital. If the child is unstable or at risk of imminent deterioration consider transferring them by ambulance.

a The dose of artesunate is 3 mg/kg for children with body weight < 20 kg and 2.4 mg/kg for children with body weight ≥ 20 kg, administered by intramuscular injection. Where artesunate is unavailable, artemether or quinine may be used [61].

Alt-text: Box 2

Box 3 **Management of child in primary or secondary care at risk for malaria and without danger signs [20] (Evidence A).**

**Table:** 

• If test is *positive* for malaria: Give recommended first line oral antimalarial. • If test is *negative* for malaria: Give appropriate treatment for other cause of fever. • If malaria test is not possible and malaria is suspected: Give recommended first line oral antimalarial. • Give one dose of paracetamol or ibuprofen in clinic for high fever (≥38.5 °C). • Advise mother when to return immediately. • Follow-up in 3 days if fever persists. • If fever is present every day for >7 days, refer for assessment.

Alt-text: Box 3

It must be stressed that accurate diagnosis of malaria using reliable diagnostic tests should be done wherever possible. Overdiagnosis can result in inappropriate use of antimalarial medication and may be associated with higher case fatality rates among patients treated for malaria who do not have the infection [60]. Nevertheless, children with malaria can deteriorate quickly. Therefore, absence or delay of parasitological diagnosis should not delay starting immediate antimalarial treatment for patients with suspected malaria, and especially for those with severe illness or other high risk groups (e.g., HIV/AIDS).

In areas where malaria is endemic it is difficult to exclude septicaemia immediately in a severely ill child and broad spectrum antibiotics should be started immediately with antimalarial treatment [61].

##### Febrile convulsions

Approximately 2–14% of young children with infection and fever may experience at least a single febrile seizure. They occur most commonly between the ages of 12 to 30 months, and are uncommon after the age of 4 years [62,63]. The exact pathophysiology is uncertain, but seizures are not directly related to the magnitude of the fever, nor the rate of temperature increase. Treatment of fever does not change the risk of febrile convulsions in susceptible children [62,64,65].

The majority of febrile seizures are classified as simple (a generalised seizure occurring once in a 24 h period and lasting <10 min). Simple febrile convulsions are not in themselves harmful, and are not necessarily indicative of serious infection [62]. Most children who have experienced a simple febrile seizure will gradually return to a normal level of alertness within an hour. If the child has a normal level of consciousness, does not appear toxic, distressed, or haemodynamically unstable and a source of the fever is evident then a period of observation without further intervention is appropriate. Features of a simple seizure and indications for referral are listed in Table 6.

Table 6 **Guidelines for referral of children with febrile convulsions [62,64–66].**

**Table:** 

**Simple seizure: May not need urgent evaluation** • Child aged between 6 months and 6 years. • Generalised tonic-clonic convulsion, no focal seizures. • Spontaneous cessation of convulsion within 10 min. • Return to alert mental status within 1 h after convulsion. • Documentation of fever (>37.5 °C). • One convulsion within a febrile illness. • Absence of pre-existing neurologic abnormality.
**Non-simple seizure: Refer for urgent evaluation** • The febrile seizure was not a simple seizure. • No cause for the fever is apparent in a young infant. • Meningitis or encephalitis cannot be excluded by history and examination. • The convulsion was prolonged (>10 min), or recovery took longer than 1 h. • The child is from a poor social setting or has limited urgent access to healthcare. • There are signs of sepsis or abnormal neurological signs, including reduced level of consciousness or excessive irritability. • The child has signs of significant malnutrition.

Alt-text: Table 6

It would be appropriate to refer every child who has a seizure at home for assessment at a hospital or by a doctor experienced in paediatric emergency care. There is likely to be significant parental anxiety associated with the event and it is important to rule out important underlying conditions, as well as to provide important information to the parents.

#### General considerations for antipyretic pharmacotherapy *(Evidence B)*

• The degree of temperature reduction in response to antipyretic medication is not predictive of presence or absence of bacteraemia [41].
• Provide general advice Box 1 for all parents, especially those who are unlikely to return for follow-up (e.g., lack of transport, parental perception that the child is not that ill, no telephone).

##### Discomfort caused by pain and fever

• Discomfort during a febrile illness is often consequent to associated pain; e.g., myalgia, abdominal pain, sore throat, ear pain, headache.
• Warning signs associated with pain and that require further investigation are listed in Table 7.

**Table 7:** Red flags in children with headache and acute abdominal pain.

Headache [67,68]	Acute abdominal pain [69,70]
1. Sudden onset of headache (first or worst ever) 2. Occipital or cluster headache 3. Early morning headache 4. Headache associated with nausea or severe vomiting, especially in early morning 5. Pain that wakes the child from sleep or occurs on waking 6. Worsening of pain in recumbent position or with cough, straining or other Valsalva manoeuvre 7. Change of the character or increased severity of headache in patients diagnosed with primary headache 8. Altered conscious state 9. Changes in mood or personality over days or weeks 10. Neurological dysfunction, cranial nerve palsies, neck stiffness, photophobia, phonophobia, projectile vomiting, positive Kernig's sign, positive Brudzinski's sign 11. Abnormal ocular movements, squint, pathologic pupillary responses 12. Visual field defects 13. Ataxia, gait abnormalities, impaired coordination 14. Seizures or fever 15. Increased head circumference 16. Papilledema 17. Poor general condition 18. Age < 5 years 19. High-risk population (e.g., patients with sickle cell anaemia, malignancy, recent head trauma, ventricular-peritoneal shunt)	1. Septic appearance (fever, tachycardia, anorexia, generally unwell) 2. Respiratory symptoms (tachypnoea, respiratory distress, cough) 3. Generalised oedema (suspect nephrotic syndrome) 4. Significant dehydration (clinically or > 5% weight loss) 5. Purpuric rash (suspect sepsis if febrile or Henoch-Schonlein purpura if afebrile) 6. Jaundice 7. Peritoneal pain (guarding, generalised or localized rebound tenderness and/or abnormal bowel sounds) 8. Faecal vomiting 9. Bilious (green) vomiting 10. Blood in stool 11. History of recent significant abdominal trauma 12. History of recent abdominal surgery 13. Abdominal pain radiating to back 14. Irreducible hernia 15. Testicular torsion (loss of the cremasteric reflex, diffuse testicular tenderness, elevated testes, and a horizontal rather than vertical position of the testes) 16. Severe or increasing abdominal pain 17. Nonmobile, or change in gait pattern due to pain 18. Abdominal distension 19. Palpable abdominal mass 20. Vaginal bleeding/discharge 21. Polyuria/polydipsia (suspect diabetes mellitus) 22. Age < 5 years (except irreducible, testicular hernia, torsion or recent abdominal injury)
• Antipyretics (ibuprofen and paracetamol) may be considered to improve comfort (with accompanying improvements in feeding activity and irritability), because they may also provide relief from pain and may reduce the risk of dehydration [3].

##### Assessing discomfort and pain in nonverbal children

• In the absence of illness that requires specific treatment, relief from distress is the primary indication for prescribing pharmacotherapy for a child with fever. Therefore it is important to assess the child's level of discomfort (Table 8) [71].

**Table 8:** Signs of distress in a child with fever and factors to consider when evaluating the level of distress [71].

Signs of distress	Factors to consider
• Change in behaviour • Change in mood • Disturbance of sleep-wake cycle • Change in feeding • Change in activity level • Reduced interest and social interaction • Reduced play • Irritability and agitation • Moaning and crying	• Age • Sex • Level of cognitive development • Cultural background • Home/residential environment • Fear and beliefs about the illness (child and parents) • Previous experiences with illness and medical care • Attitude of parents/caregivers
• Various tools exist to assess pain in children who are unable to communicate [72]. The Evaluation Enfant Douleur (EVENDOL) behavioural scale has been validated in children from age newborn to 7 years presenting with pain in the in-hospital and out-of-hospital emergency settings (Table 9) [[72], [73], [74]]. The score on EVENDOL ranges from 0 to 15 and it remains a useful tool despite presence of fever, hunger or anxiety. The treatment threshold is 4/15 [73].

**Table 9:** EVENDOL Pain Scale [73] (Score ranges from 0 to 15. Treatment threshold is 4/15).

Behavioural and environmental expressions	Sign absent	Sign weak or transient	Sign moderate or present about half the time	Sign strong or present almost all the time
*Vocal or verbal expression:* Cries, screams, moans, complains of pain	0	1	2	3
*Facial expression:* Furrowed forehead, frown, furrowed or bulging brow, tense mouth	0	1	2	3
*Movements:* Restlessness, agitation, rigidity, muscular tension	0	1	2	3
*Postures:* Unusual and/or antalgic posture, protection of the painful area, immobility	0	1	2	3
*Interaction with the environment:* Can be comforted, interested in playing, interacts with people	**Normal** 0	**Low** 1	**Very low** 2	**Absent** 3

Reprinted with the authors' permission. Copyright EVEDOL Group.

#### Antipyretic medication

• Antipyretics should be used to make the child more comfortable and not used routinely with the sole aim of reducing the temperature [2,3,21,71].
• When antipyretics are not indicated at time of consultation, one or two days of antipyretic medication may be prescribed for use at home should it become necessary (see Box 4, Box 5).
  Box 4 **General advice about fever for parents (and other caregivers).**
  **Table:** 

**1. Reassure parents with anxiety about the child's fever.** • Explain the nature of the child's illness. • Fever is not an illness, but a beneficial response of the body to illness. Most fevers are of short duration and are not harmful. • Children with fever are not at increased risk of seizures, dehydration, brain damage or death. • Body temperature during fever normally fluctuates and the fever will run its course. • The fever will return until the illness is better and strict control of fever is never required. • Fever after vaccination is normal and not harmful. Most vaccine-related fevers are detectable 10 to 20 h after vaccination and the duration of fever is usually <12 h [109]. • If an antibiotic is not indicated, explain the reasons why and harms associated with prescribing an antibiotic. **2. Advise the parent on management of fever at home (**Box 5**).** **3. Give clear instructions on how to administer medication.** • Correct dose; how to measure the dose; how often to administer a dose. • Warn parents not to exceed the prescribed dose or dosing interval. • Unless the medicine comes with a measuring device, caregivers should be provided with an appropriate syringe or measuring spoon whenever medicine for a child is dispensed. **4. Advise parents on the correct use of antipyretic medication.** • Antipyretics are used to make the child more comfortable by reducing symptoms. • Antipyretics are not used routinely with the sole aim of reducing the temperature and will not reduce body temperature to normal. • Antipyretics will not cure the illness. • Antipyretics do not prevent febrile convulsions and should not be used specifically for this purpose. • Antipyretic medication starts to work within 1–3 h, but will not bring body temperature to normal unless the fever was low to start with. • If the child vomits immediately after taking a dose of medicine, another dose may be given, but care must be taken not to overdose. For children who are vomiting intermittently, suppositories may be used instead of oral medication. Suppositories and oral medication of the same type should not be used together. • Avoid combination products and ‘cough and cold medicines’, which complicate dosing and may increase the risk of overdose and side effects. **5. Provide written instructions about follow-up.** • When to come back; what to do if the child's condition gets worse (see Box 1).
  Alt-text: Box 4
  Box 5 **Advice for parents (and caregivers) about management of fever at home (Evidence C).**
  **Table:** 

Do	Don't
**Do** encourage the child to drink fluids regularly (breast milk is best for breast feeding children). **Do** administer antipyretic medication if the child is distressed. **Do** make sure the dose is correct based on the weight of the child. **Do** shake the bottle before pouring a medicine. **Do** use a syringe or medicine measure spoon to administer medicines. **Do** store all medicines out of the reach of children. **Do** store all medicines according to the manufacturer's instructions and below 25 °C, out of direct sunlight, with the cap tightly closed. Do not keep medicines in the car or bathroom cabinet. **Do** seek further medical advice if the fever does not get better within 48 h, or if the child's condition gets worse (Box 1).	**Don't** apply tepid sponging. Sponging the skin with cool water may briefly reduce the temperature of skin being sponged, but it does not reduce inflammation or affect the cause of fever and therefore has no significant lasting effect on body temperature. **Don't** over-dress or under-dress the child, or wrap the child in heavy blankets. **Don't** exceed the maximum recommended doses of antipyretic medication per 24 h. **Don't** measure medicine using a household teaspoon or tablespoon – use only the measuring device provided. **Don't** allow children to drink medicines straight from the bottle. **Don't** administer another dose of antipyretic medication immediately if the temperature does not come down after one dose. Wait for the appropriate dosing interval to pass and only give another dose at the correct time. **Don't** wake a sleeping child just to administer antipyretic medication. **Don't** give antipyretic medicine to the child for longer than 2 days without consulting a doctor. **Don't** give a child antipyretic medication before or immediately after vaccination.
  Alt-text: Box 5
• Antipyretic medications do not reduce the risk of febrile seizures and should not be prescribed for this indication [21,64,65].
• The use of antipyretic medication and attention to the fever must not detract from monitoring the child's activity and level of consciousness (as an indicator of worsening illness) and attention to adequate hydration.
• Both paracetamol and ibuprofen are safe and effective for short-term use in children and are the drugs of choice to manage fever [3,21,[75], [76], [77], [78], [79], [80], [81], [82], [83], [84], [85], [86]]. *(Evidence A)*
• The practices of combining or alternating paracetamol and ibuprofen have limited value and are not recommended [82,[87], [88], [89]]. *(Evidence B)*
• Suppositories may be useful when the child is unable to take oral medication. However, especially in younger children, absorption and bioavailability may be more variable than with oral medication [[90], [91], [92], [93], [94], [95], [96], [97]].
• IV paracetamol is an equally effective alternative to oral paracetamol when the child cannot take medicine by mouth [[98], [99], [100], [101], [102]].
• Mefenamic acid is registered for use from 6 months of age and may be a second-line alternative NSAID to ibuprofen. However, in comparison to paracetamol and ibuprofen, there is limited safety data for mefenamic acid use in children. Care should be taken to avoid overdosing, which has been associated with adverse effects. The recommended dose is 6.5 mg/kg body weight, not more than three times daily. *(Evidence B)* [103,104]
• Dosing of antipyretic medication in children should be accurately based on body weight and should not be estimated (Table 10, Table 11, Table 12). For accurate dosing, liquid medicines should be administered with a syringe. If a spoon is used, it should be a medicine measure spoon (not a teaspoon).

**Table 10:** Dose of antipyretic medication for infants and children older than 3 months of age [3,22] (*Evidence B*).

	Oral dose	Dose frequency	Maximum daily dose^a^
Ibuprofen	10 mg/kg body weight	every 6 h as necessary	40 mg/kg
Paracetamol	15 mg/kg body weight (up to 1 g)	every 6 h as necessary	90 mg/kg (4 g in total)

a Do not exceed this dose within a 24 h period.

**Table 11:** Dosing by body weight for oral liquid: Ibuprofen 100 mg/5 mL every 6 h.

Body weight (kg)	Using a syringe (mL)
6	3.0
9	4.5
12	6.0
15	7.5
18	9.0
21	10.5
24	12.0
27	13.5
≥30	15.0

**Table 12:** Dosing by body weight for oral liquid: Paracetamol 120 mg/5 mL every 6 h.

Body weight (kg)	Using a syringe (mL)
6	3.5
9	5.5
12	7.5
15	9.5
18	11.0
21	13.0
≥24	15.0
• Do not administer paracetamol or ibuprofen more frequently than every 6 h (a total of four doses in a 24 h period).
• Aspirin should not be used for children or adolescents ≤18 years of age. It has been associated with Reye's syndrome and may increase the risk of bleeding in infections with bleeding risk [22]. *(Evidence A)*
• Both paracetamol and ibuprofen have been associated with increased risk of bronchospasm in a very small percentage of predisposed children with asthma [105]. Care should be taken when using antipyretic medication for these children.
• The use of NSAIDs has been associated with an elevated risk of severe skin and soft tissue infections in patients with varicella zoster virus infection [[106], [107], [108]]. Therefore, paracetamol is recommended as the antipyretic of choice in children with chicken pox.
• Medicines containing combinations of NSAIDs, paracetamol, codeine and/or antihistamines should not be used in children. *(Evidence B)*

#### Vaccination and fever *(Evidence B)*

• Fever and a local inflammatory reaction (pain, swelling, redness) are normal responses to vaccination and are not harmful. Parents should be warned that fever may occur after vaccination.
• Time to onset, maximum temperature and duration of fever after vaccination is variable, depending on the type of vaccine [109].
• Administration of antipyretic drugs before or at the time of vaccination, or in the first 6 to 8 h after vaccination, is associated with reduced antibody responses to vaccine antigens [[110], [111], [112], [113]].
• Antipyretic medication should not be administered with or immediately after vaccination either as a treatment for a local inflammatory reaction or fever, or prophylactically to prevent a local inflammatory reaction or fever [23].
• Antipyretics may be considered to make the child more comfortable in the event of complications associated with vaccination, such as pain or fever during the days after vaccination, cellulitis or systemic complications [23,114].

### Advice for parents caring for the child at home

Parents require clear instructions on how to manage and monitor the child at home. Reassure them that fever itself does not necessarily require treatment, but is a symptom of an illness requiring a diagnosis to direct specific and appropriate treatment (Box 4, Box 5).

### Conclusion

Although fever in children is often benign and self-limiting, the cause of the fever can present a diagnostic challenge to the healthcare provider. However, with timeous identification, the child at risk of serious illness can be appropriately managed and quickly referred to hospital if necessary.

When the risk of serious illness is low, parents and caregivers need to be reassured, and the child should be managed appropriately at home with antipyretic medication, if it is indicated, to make them more comfortable. Clear instructions to advise parents and caregivers about when to seek further care for their child will help to reduce the morbidity associated with childhood illnesses.

We hope this document will be of value in clinical practice and that the tables and other important information presented herein may be extracted as appropriate to compile more simple reference guides to suit the activities of individual healthcare workers and clinics across the sub-Saharan Africa region.

## CRediT authorship contribution statement

RG and DW wrote the initial draft of the document. Thereafter, the authors contributed equally to drafting the work or revising it critically for important intellectual content. All authors agreed to be accountable for all aspects of the work.

## Declaration of competing interest

The development of this document was supported by an unconditional grant from Reckitt Benckiser. The sponsor did not participate in the development or writing of the document.

RJG has been a member of the speakers bureau for Reckitt Benckiser, GlaxoSmithKline and Adcock Ingram. DW is a medical writer and reports personal fees from Reckitt Benckiser during development of this work; personal fees from Adcock Ingram, Astra Zeneca, Cipla, Fresenius Kabi, Litha, MSD, MundiPharma, Mylan, Novartis, Novo Nordisk, Pfizer, Pharma Dynamics, Reckitt Benkiser, and Sanofi-Aventis outside the submitted work. PMJ has been a member on the speaker bureau for Reckitt Benckiser, MSD and Pfizer. RM has been a member of the speaker's panel for Pfizer Consumer, Astra Zeneca, Reckitt Benckiser, Adcock Ingram, Mylan and Aspen Pharmacare. JM has been an advisor to, and received payment for travel from, Reckitt Benckiser. MW is an editor of the African Journal of Emergency Medicine. MW was not involved in the editorial workflow for this manuscript. The African Journal of Emergency Medicine applies a double blinded process for all manuscript peer reviews. The authors declared no further conflict of interest.
